# Neck-specific strengthening exercise compared with sham ultrasound when added to home-stretching exercise in patients with migraine: study protocol of a two-armed, parallel-groups randomized controlled trial

**DOI:** 10.1186/s12998-020-00313-w

**Published:** 2020-05-19

**Authors:** Mariana Tedeschi Benatto, Lidiane Lima Florencio, Marcela Mendes Bragatto, Fabíola Dach, César Fernández-de-las-Peñas, Débora Bevilaqua-Grossi

**Affiliations:** 1 Department of Health Sciences – Ribeirão Preto Medical School, University of São Paulo, 3900, Bandeirantes Avenue - Monte Alegre, Ribeirão Preto, SP 14049-900 Brazil; 2 Department of Physical Therapy, Occupational Therapy, Rehabilitation and Physical Medicine, Universidad Rey Juan Carlos, Alcorcón, Spain; 3 Department of Neurosciences and Behavioral Sciences – Ribeirão Preto Medical School, University of São Paulo, Ribeirão Preto, SP Brazil

**Keywords:** Cervical exercises, Migraine disorders, Electromyography, Neck pain

## Abstract

**Background:**

Migraine is a highly disabling condition and pharmacological treatment is the gold standard. However, several patients have also positive responses to the application of different manual techniques and therapeutic exercises in terms of reducing the intensity and frequency of migraine attacks. Nevertheless, the effects of a neck-specific exercise program have not yet been evaluated in these patients.

**Objective:**

To determine the effectiveness of a neck-specific exercise program in reducing the intensity and frequency of migraine attacks as compared to a sham ultrasound group.

**Methods:**

A two-armed, parallel-groups randomized controlled trial with 3 months of follow-up will be conducted. 42 individuals, both genders, aged between 18 and 55 years old with a medical diagnosis of migraine will be included. The intervention group will perform a protocol consisting of exercises for strengthening the muscles of the cervical spine. Participants within the sham ultrasound group will receive detuned ultrasound therapy in the upper trapezius muscle. Both groups will receive a weekly session for 8 weeks. The efficacy of each intervention will be measured by the frequency and intensity of migraine at a 3-months follow-up.

**Trial registration:**

This study was registered under access code RBT-8gfv5j in the *Registro Brasileiro de Ensaios Clínicos* (ReBEC) in November 28, 2016.

**Conclusion:**

This study will aim to determine the efficacy of a neck-specific exercise program in reducing the frequency and intensity of migraine attacks. If the results show that a neck-specific exercise program is effective in reducing the frequency and intensity of migraine attacks, therapists will have a low cost and easily applicable tool to treat migraine.

## Introduction

Migraine is classified as a chronic [1] and disabling disease [2] characterized by recurrent headache attacks lasting from 4 to 72 h, with a unilateral location and pulsating quality. Migraine attacks range from a moderate to severe intensity that increases with daily routine, physical activity, and can be accompanied by nausea and/or photophobia and phonophobia [3]. In addition to these features, neck pain is quite common [4] and is associated with a worse prognosis for the disease [5].

Pharmacologic treatment is currently the gold standard for migraine management [6]. However, a multidisciplinary approach is also recommended [7] and manual techniques and therapeutic exercises may be valuable adjunct treatments as they help to reduce symptoms, prevent migraine attacks [8], and result in a relevant clinical reduction of the frequency and intensity of migraine attacks while increasing patient satisfaction with the treatment [9].

Among the various manual technique interventions, aerobic exercises in combination with physical and psychological interventions result in a reduction in the duration of migraine attacks [8]. It is known that aerobic exercises were as effective in reducing the frequency of migraine attacks as standard pharmacological treatment with topiramate and tricyclics [10]. In addition, manual therapy interventions targeting the craniocervical region have also demonstrated beneficial results, such as improvement in the quality of life, reduction of pain intensity, reduction in related disability, and a reduced frequency of migraine attacks [11–14]. However, despite evidence showing that different manual techniques and therapeutic exercises in the craniocervical region are effective in migraine management [8–14], to date, the effects of cervical muscle strengthening exercises on this population are unknown [15].

In individuals suffering from neck pain, neck-specific exercises have been shown to be effective in reducing neck pain [16–18], improving performance in the craniocervical flexion test [16], and strengthening the cervical deep flexor and extensor muscles [19–21]. Considering that individuals with migraine also exhibit cervical dysfunctions [8, 22–27], we believe that, similar to what has been observed in subjects with neck pain [28], individuals with migraine may also benefit from these types of exercises. The hypothesis of this study is that the strengthening exercises have greater improvement than sham ultrasound in reducing migraine frequency and intensity when practiced in combination with home stretching exercises.

### Primary and secondary objectives

The primary objective of the current study is to determine the efficacy of a neck-specific strengthening exercise program in reducing the frequency and intensity of migraine attacks in subjects with migraine, compared with the sham ultrasound group. We also intend to determine whether craniocervical exercises influence the intensity of migraine attacks, migraine-related disability, concomitant neck pain, and the quantitative variables, including neck range of movement, pressure pain threshold, motor control of the deep flexor muscles, neck muscle strength, and resistance of cervical musculature.

## Methods

### Design

A two-armed, parallel-group randomized controlled trial designed according to the CONSORT guidelines [29].

### Ethics approval

The protocol has been approved by the local ethics committee (no. 6862/2016) and registered under access code RBR-8gfv5j in the Registro Brasileiro de Ensaios Clínicos (ReBEC).

### Setting of the study

The study protocol described here was designed according to the recommendations of SPIRIT [30]. Given that this protocol will be applied in individuals with migraine, our selection of criteria for inclusion, exclusion, and choice of outcomes was also guided by the suggestions of the International Headache Society [31].

The study will be conducted in a single center, located at the University of São Paulo, in Ribeirão Preto, Brazil, and will include three main researchers, two of whom will be responsible for data collection and one of whom will be responsible for the treatment in both groups. In addition, a team of four more researchers will be responsible for screening volunteers.

### Participants

Volunteers for the study will be included if they are between 18 and 55 years old, are of either sex, have been diagnosed with migraine according to the third edition of the International Classification of Headache [3], and have exhibited at least 3 days with migraine attacks in the previous month [31]. The inclusion and exclusion criteria are fully described in Table 1.

**Table 1:** Inclusion and exclusion criteria

Inclusion	Exclusion
18–55 years old	Another type of headache
Both sexes	Medication overuse headache
Diagnosis of migraine	History of trauma in the cervical and/or face
Minimum of 3 days with migraine/month	Diagnosis of hernia or disk degeneration in the cervical region
	Systemic diseases^a^
	Pregnancy
	Anesthetic block in the three months prior to the selection process
	Have received manual techniques or therapeutic exercises in the last year in craniocervical region

^a^e.g.: fibromyalgia, diabetes, peripheral neuropathies

### Recruitment

Volunteers will be recruited from the local population by announcements. The study will be released on social media (Facebook®, Instagram®, and local university radio), and those who apply will be referred to a neurologist with at least 5 years of experience in the diagnosis of headache and migraine, who will perform the migraine diagnosis. Those participants who meet all eligibility criteria will be invited to participate in the trial.

### Study schedule

#### Screening

A prescreening will be conducted among individuals who expressed an interest in the study on social media. The basic criteria of their migraine will be queried (e.g., location, intensity, and type of headache), and those who apparently fit the characteristics of migraine will be referred to the neurologists. After confirming the diagnosis of migraine and the inclusion criteria, the volunteer will be invited to participate in the study. Those who accept the invitation will receive all pertinent information about the study and begin to fill a paper headache diary [9]. After 30 days, the volunteer will undergo evaluation 1, which will consist of the application of questionnaires and a physical examination, and at the end of evaluation 1 randomization will occur. Depending on patient availability, treatment will begin in the next few days. Follow-up will occur 1, 2, and 3 months after the end of treatment.

**Fig. 1:**
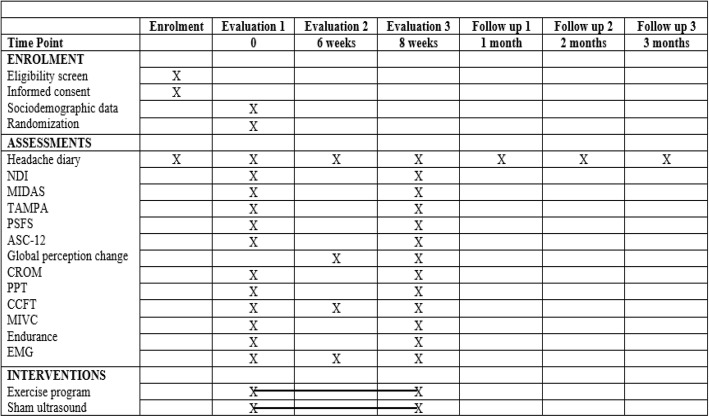
Study schedule summary *NDI* Neck Disability Index; *MIDAS* Migraine Disability Assessment; *TAMPA* Tampa Scale for Kinesiophobia; *PSFS* Patient-specific Functional Scale; *ASC-12* 12 item Allodynia Symptom Checklist; *CROM* Cervical range of motion; *PPT* pressure pain threshold; *CCFT* craniocervical flexion test; *MIVC* maximal isometric voluntary contraction; *EMG* electromyography

The study design, which covers the screening, evaluation, intervention, and follow-up procedures is summarized in Fig. 1.

#### Randomization and blinding

Participant allocation will be determined in sequential order, randomized by Excel®. Blinding will be ensured by giving the participant a brown and opaque envelope containing their group assignment, which will be opened in front of each participant. The envelopes will be prepared in advance by an independent researcher; envelopes will be opened in front of each participant. A single therapist, blinded to the results obtained during evaluations, will be responsible for all the interventions in both groups. Similarly, the therapist responsible for data collection will be blinded to the treatment allocation group (intervention or sham ultrasound).

### Interventions

This study will be performed to compare a neck-strengthening exercise protocol with a sham ultrasound group in migraine subjects. In both groups, there will be a combination of home stretching exercises. The interventions in both groups will last for 2 months. After completion of treatment, patients of both groups will be followed up by phone at 1, 2, and 3 months.

**Fig. 2:**
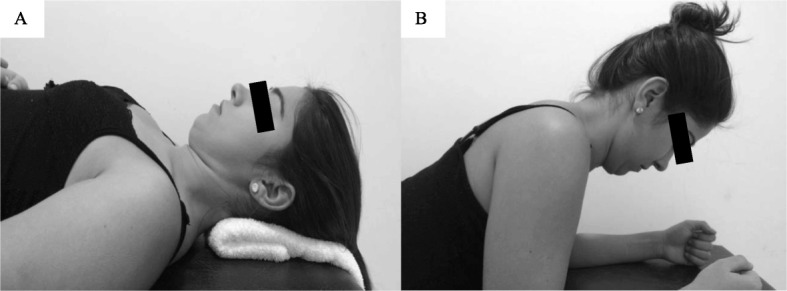
First stage of neck-specific exercise program

**Fig. 3:**
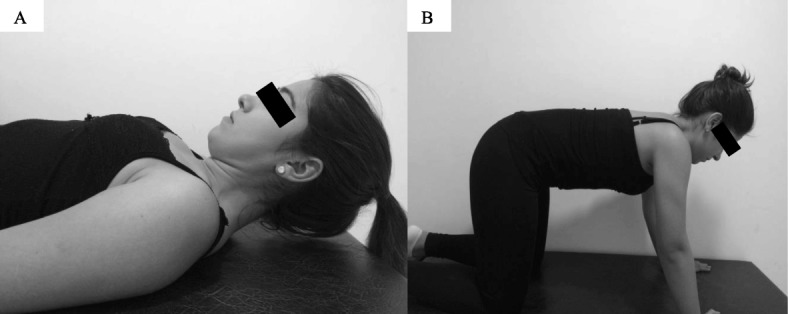
Second stage of neck-specific exercise program

Intervention group (IG): the 8-week exercise program to be used in this study will be based on the protocol described by Falla et al. [28], which consists of specific exercises to strengthen the flexor and extensors muscles of the neck. Participants will receive instructions and guidance from a trained therapist for 30 min once a week. The protocol consists of two stages:

- *Stage 1:* The first stage, of 6-week duration, will focus on incremental craniocervical flexion in a relaxed, supine position and craniocervical extension, flexion, and rotation in a prone-on-elbows position while maintaining the cervical spine in a neutral position (Fig. 2) [28]. The initial proposal for the deep flexors and extensors of the cervical spine will be 2 sets of 10 repetitions. However, for the deep flexor muscles, the movement should be initially sustained for 10 s. If the participant cannot perform the targeted program, the number of sets, repetitions, and time of sustentation (for the deep flexors) will be redefined. According to the tolerance of the patient, the sets, repetitions, and time of sustentation will be increased weekly [28].
- *Stage 2:* The second stage, of 2-week duration, consists of both flexor and extensor exercises with higher load, using head weight as the load [28]. A neck flexion associated with a head flexion will be performed with the participant in the supine position, removing the head from the stretcher. For the extension exercise, while performing the neck extension the patient will be instructed to maintain the cervical spine in the neutral position, and the body in the position of 4 supports (Fig. 3). Each of the exercises will be for 3 sets of 15 repetitions at a time over the course of 2 weeks [28].

Participants will be instructed to perform the same exercises at home, twice a day, for 10–20 min a day. They will receive a guideline book containing a neck-specific exercise program, including neck muscle stretching exercises.

Sham ultrasound group (USG): participants in this group will have meetings once a week for 8 weeks. The application of disconnected of therapeutic ultrasound (US) will be performed bilaterally in the middle portion of the upper trapezius. The application time will be around 9 min for each side, totaling approximately 20–25 min per session. Participants will also receive a guideline book, containing only cervical spine stretching exercises.

### Outcomes

The primary outcomes of this study will be the frequency and intensity of migraine. The frequency of headache attacks data will be collected by means of the headache diary, which will be delivered monthly to the patient. The frequency of migraine will be computed and analyzed in terms of days-of-pain per month. The migraine intensity will be also collected in the headache diary. The volunteers will classify the pain according to the numerical pain scale (NPS; 0–10) [32], and for the analysis of these data an average will be used. The secondary outcomes will be as follows: global perception of change, migraine-related disability, neck pain, cervical range of motion, pressure pain thresholds, muscle performance, observed by muscular strength, the cranio-cervical flexion test (CCFT), and the endurance of cervical musculature, in addition to the electromyographic parameters used in these tests.

### Process variable

All volunteers will meet weekly with the responsible therapist. During all meetings, the therapist will ask, and note, if the volunteers: are doing the home care. If volunteers get additional care, the therapist will be informed and this information will be noted. As well as the use of non-prescribed medications.

### Data collection

All data, except the electromyographic signals, will be collected and noted by the evaluators on paper records specific to the evaluations. Electromyographic data, in turn, will be collected and automatically stored in individual file folders in a computer used exclusively for the study. After the conclusion of the data collection, the data will be transcribed into Excel® spreadsheets by an examiner not involved with the data collection or treatment, using codes to preserve volunteer identification. The frequency and intensity of migraine attacks will be recorded in paper-pain diaries. After 30 days, the diaries will be given to the therapist responsible for the collections to transcribe the data into Excel® spreadsheets (Fig. 1).

### Methods for data collection

#### Questionnaires

- *Neck Disability Index* (NDI): a valid instrument with good responsiveness [33], is the most recommended means to evaluate the functional disability associated with neck pain [34].
- *Migraine Disability Assessment* (MIDAS): a valid and reliable questionnaire used to assess the functional disability related to migraine [35, 36].
- *Tampa Scale for Kinesiophobia* (TAMPA): a 17-item scale originally developed to measure the fear of movement related to chronic lower back pain; however, it is also used to measure the fear of movement in different parts of the body and presents a good applicability psychometric for the cervical region [37].
- *Patient-Specific Functional Scale* (PSFS): a valid, reliable, and sensitive scale used to assess functional changes over time in patients with musculoskeletal disorders [38].
- *12 item Allodynia Symptom Checklist* (ASC-12): a reliable instrument to determine the presence and severity of cutaneous allodynia [39].
- *Global Perception of Change:* a one-dimensional scale in which individuals rate their improvement associated with an intervention on a scale of seven items [40].

#### Cervical range of motion

The cervical range of motion for flexion, extension, lateral-flexion, and rotation of the neck will be performed using the Cervical Range of Motion (CROM) (Performance Attainment Associates, Roseville, MN, USA). Two trials of each movement will be performed with the volunteer in a chair in their usual sitting position [22].

#### Pressure pain thresholds

Pressure pain thresholds will be assessed by using a digital manual dynamometer (DDK-10 Kratos®). A pressure of approximately 0.5 kg/cm^2^/s will be applied, with optimal positioning of the device perpendicular to the evaluated anatomical surfaces [24]. In addition, a digital metronome with a frequency of 1 Hz will be used to provide audio feedback and standardization of the pressure application speed [41]. Pressure pain threshold will be assessed randomly and repeated twice, bilaterally, over the upper trapezius, the sternocleidomastoid, the suboccipital, temporal, frontal, and levator scapulae [24, 42–44].

#### Craniocervical flexion test (CCFT)

The muscular performance of the cervical deep flexors will be evaluated by using the CCFT, a neuromuscular low-load test used to evaluate the activation and endurance of the deep flexors [45]. The test is performed with the subject supine with a pressure biofeedback unit (Stabilizer, Chattanooga, South Pacific; USA) placed behind the neck and inflated to 20 mmHg. The subject is instructed to perform a gentle and slow head-nodding action of craniocervical flexion over five incremental stages of increasing range (2 mmHg at each stage), with each stage maintained for 10 s. Subjects will perform two repetitions of 2 s at each stage in order to familiarize themselves with the task. During this familiarization test, signs of inappropriate performance, such as head retraction, head lift, or difficulty relaxing after the contraction are discouraged. Subjects then perform the CCFT holding the target level for 10 s with a 30 s rest period between levels without any control of potential compensations. The test is interrupted following the identification of any compensation, such as simultaneous palpable contraction of superficial flexors, and the latest stage performed without any compensation, will be registered as the targeted level [45].

#### Muscle strength during the maximal isometric voluntary contraction (MIVC)

**Fig. 4:**
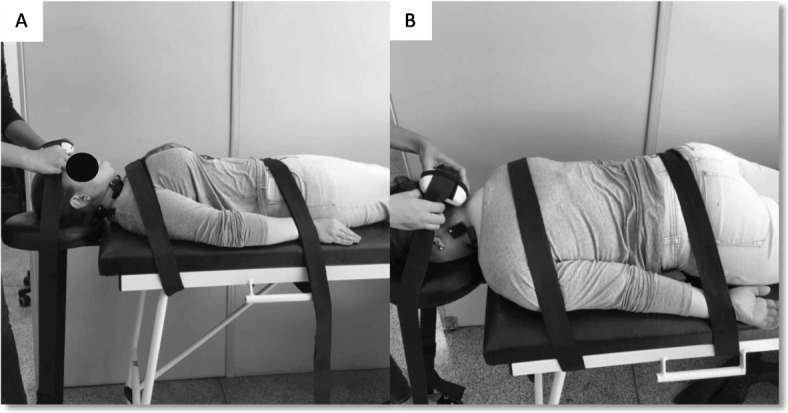
Muscle strength during the maximal isometric voluntary contraction. (**a**) cervical flexors; (**b**) cervical extensors

The measurement of muscle strength during a MIVC will be performed according to the protocol described by Carnevalli et al. [46] A manual dynamometer attached to a non-elastic belt will be used (Lafayette Instrument Company®, model: 2201163, Lafayette, IN, USA). For measurement of flexion force, the patient will be placed in the supine position and will have their pelvis and trunk stabilized by non-elastic belts; the dynamometer will be positioned in a midline on the frontal bone. The measurement of cervical extensor strength will occur with patients in the prone position and stabilization at the same points as the flexor test; the dynamometer will be positioned in a midline over the occipital bone. For both measurements, 3 trials will be performed with contractions sustained for 3 s. Rest of 30 s will be allowed between repetitions, and 2 min between muscle groups (Fig. 4).

#### Endurance test of flexor and extensor muscles of the cervical spine

**Fig. 5:**
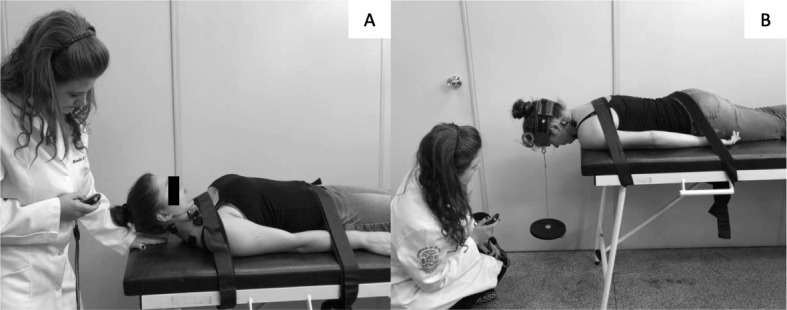
Endurance test of flexor (**a**) and extensor (**b**) muscles of the cervical spine

To verify the endurance of the cervical flexor muscles, subjects will be placed in the supine position with their head resting on the hand of the therapist in a neutral position. At the beginning of the test, the volunteer should perform a head and neck flexion by lifting their head up from the stretcher; the holding time will be registered in seconds and only one repetition will be performed. The test will be interrupted if the therapist notes the presence of pain or fatigue, or if the head of the volunteer touches the hand of the therapist [47]. For the neck extensors, the volunteer will be placed in a prone position with the head resting on a removable support of the stretcher; stabilization will occur at the T6 level by a non-elastic belt. To measure the degrees of displacement of the head a CROM device will be used and a weight of 2 kg will be added, attached to a non-elastic band positioned on the head of the patient. The support will be removed, and the patient should keep their cervical spine in the neutral position. The holding time will be registered in seconds and only one repetition will be performed. The test will be stopped in case of pain, fatigue, or if the head inclination varies more than 5° (Fig. 5) [47].

#### Surface electromyography

Activation of the cervical muscles will be recorded by surface electromyography during the CCFT, MIVC, and muscle endurance test. Wireless surface sensors (TrignoTM Wireless System, Delsys Inc., Boston, MA, USA) will be firmly fixed with adhesive tape, bilaterally over the following: 1, sternal head, 1/3 distal of the muscle belly of the sternocleidomastoid muscle (SCM) [48]; 2, the anterior scalene muscles, third portion of the muscle belly, parallel to the clavicular head of the SCM [48]; 3, the muscle belly of the splenius capitis muscles [49]; and 4, midpoint between the acromion and C7, parallel to the fibers of the descending upper trapezius muscle [50].

The EMG Works*®* version 4.1.5 (Delsys Inc.) software will be used to acquire and display the electromyographic signals during the acquisition. Raw electromyographic signals will be processed by a custom MATLAB*®* routine responsible for filtering them at a frequency band of 20–500 Hz, using a 4th order Butterworth filter, and the root mean square (RMS) of the filtered signal for the acquisition time will be calculated in each test.

### Statistical analysis

All statistical analyses will be performed by a professional statistician and they will be blinded to the allocation groups. An intention-to-treat analysis will be performed in order to verify the effect on the outcomes. Intention-to-treat analysis is the most indicated statistical analysis method in randomized clinical trials because it allows verification of the real efficacy of the intervention, and avoids an overly optimistic estimate that may result from removal of the non-adherent volunteers [51]. Mixed linear regression models will be performed for the primary and secondary outcomes to estimate between-group and within-group comparisons. “Time” and “group” will be considered as fixed effects and participants as a random effect (random intercept model). Baseline will be used as a covariate and the term “interaction time by group” will be included in the model to evaluate the interaction effect of differences between groups at each follow-up and within-group differences over time. Additionally, post hoc Bonferroni correction will be performed for group comparisons at each follow-up in the case of a significant “time” X “group” interaction.

In addition, to determine the clinical relevance of between-groups differences, Minimal Clinically Important Difference (MCID) calculations will be performed for the variables that do not have the MCID described in the literature, and the Effect Size (ES) will be calculated based on the distribution methods. Differences will be considered clinically relevant when they overcome the MID combined with an ES > 0.4, following the criteria suggested by Armijo-Olivo et al. [52]

For the missing data, as recommended by CONSORT [29], imputation occurs through a mixed effect regression model*.*

### Sample size

A sample size of 21 individuals on each group was the result of a calculation in which a level a significance (α) of 0.025 was adopted, with a power of 80%, a between-groups difference to be detected of 3.3 (SD = 3.1) days of headaches per month, and considering a loss of 20% at follow-up. The mean and standard deviation used in the sample size calculation were based on the reduction of the frequency of a gold standard pharmacologic treatment group for migraine management from a previous clinical trial [9]. For the primary outcome “headache intensity”, adopting the same parameters as the previous calculation, a sample size of 20 would be necessary to detect a between-groups difference of 1.52 (SD = 1.37) in the NPS, based on data from 4-week pharmacological treatment [53].

## Discussion

### Potential impact and significance of the study

The neck-specific exercise program used in this trial is currently a widely discussed component of the rehabilitation for patients with neck pain. It has been recommended due to its efficacy in decreasing the intensity of neck pain and improving the quality of life [17, 18]. However, to date, this study would be a pioneer in verifying the efficacy of this protocol in patients with migraine. We believe that individuals may benefit from this protocol, as patients with migraine have several musculoskeletal disorders. If the effect of this exercise program for patients with migraine is similar to that observed in patients with neck pain, it would be possible to reduce the impact of migraine by reducing the frequency and the intensity of migraine attacks, which is the main goal of the multi-professional team in the management of migraine [7].

### Strengths and weaknesses of the study

Strengths of the study include the design and the novelty of investigating whether a neck-specific strengthening exercise program might reduce the frequency and intensity of migraine attacks. The gold-standard treatment for migraine is pharmacological. However, manual techniques and therapeutic exercises have shown satisfactory results in reducing migraine attacks and improving quality of life. We recognize that the application of an exercise program alone is a limitation of this study, because it may not represent common clinical practice. However, from a scientific perspective, the effects of this protocol should be specifically determined before combining it with other interventions.

We can cite as other limitations: no long-term follow-up, attention bias from the differences between groups in the time spent with a therapist, long home exercise assignments, only partial blinding of participants (they can distinguish treatments, but they are blinded to sham ultrasound), the estimates of the mean and standard deviation used in the sample size calculation came from a different study population, and adverse events will not be collected.

Finally, although we will attempt to make the number of meetings the same when inserting the sham group with ultrasound, patients who will receive this placebo may have the feeling of greater attention because they will receive a type of hands on therapy. In addition, patients in the sham group may have a greater perception of improvement than the intervention group. However, implementing a placebo group that received another type of exercise would also be controversial because the effects of non-specific exercises on migraine are already known.

### Contribution to the physical therapy profession

We believe that the current trial will bring innovative and unprecedented results to improve the management of individuals with migraine. If we observe that the neck-specific exercise program is effective in reducing the frequency and intensity of migraine attacks, professionals will be offered a low-cost and easily applicable therapeutic option.

## Data Availability

The datasets used and/or analysed during the current study are available from the corresponding author on reasonable request.
